# Correlation of cardiovascular risk predictors with overweight and obesity in patients with familial hypercholesterolemia

**DOI:** 10.3389/fcvm.2022.1026243

**Published:** 2022-11-28

**Authors:** Yaodong Wang, Jinchun He

**Affiliations:** ^1^Laboratory Medicine Center, Lanzhou University Second Hospital, Lanzhou University, Lanzhou, Gansu, China; ^2^The First School of Clinical Medicine, Lanzhou University, Lanzhou, Gansu, China; ^3^Department of Laboratory Medicine, The First Hospital of Lanzhou University, The First School of Clinical Medicine, Lanzhou University, Lanzhou, Gansu, China

**Keywords:** hypercholesterolemia, cardiovascular, obesity, atherogenic, correlation

## Abstract

**Purpose:**

We aimed to analyze the correlation between overweight and obesity-related indicators and cardiovascular risk predictors in patients with familial hypercholesterolemia (FH) and to evaluate their mutual predictive properties.

**Methods:**

A total of 103 patients with FH included from 2004 to 2017 were retrospectively analyzed. Pearson correlation analysis and multiple linear regression analysis were used to assess the correlation between overweight and obesity-related indicators and cardiovascular risk predictors in FH patients. Subject operating characteristic (ROC) curve was used to analyze their reciprocal predictive performance.

**Results:**

(1) Atherogenic index of plasma (AIP) (β = 0.020) and ApoB/ApoA1 Ratio (BAR) (β = 0.015) were independently correlated with body mass index (BMI) (*P* < 0.05); AIP (β = 1.176) was independently correlated with waist-to-hip ratio (WHR) (*P* < 0.01); AIP (β = 1.575), BAR (β = 0.661) and atherogenic coefficient (AC) (β = 0.427) were independently correlated with waist-to-height ratio (WHtR) (*P* < 0.05). (2) The area under the ROC (AUC) for overweight corresponding to AIP, BAR, and AC were 0.695 (95% *CI* = 0.593–0.797, *P* < 0.01), 0.660 (95% *CI* = 0.555–0.766, *P* < 0.01), and 0.632 (95% *CI* = 0.525–0.740, *P* < 0.05), respectively; and AUCs for central obesity corresponding to AIP, BAR and AC were 0.757 (95% *CI* = 0.656–0.857, *P* < 0.001), 0.654 (95% *CI* = 0.536–0.771, *P* < 0.05) and 0.651 (95% *CI* = 0.538–0.764, *P* < 0.05), respectively. The AUCs for moderate risk of AIP corresponding to BMI, WHR, and WHtR were 0.709 (95% *CI* = 0.608–0.811, *P* < 0.001), 0.773 (95% *CI* = 0.678–0.867, *P* < 0.001), 0.739 (95% *CI* = 0.641–0.836, *P* < 0.001), respectively, and BMI, WHR and WHtR corresponded to an AUC of 0.691 (95% *CI* = 0.585–0.797, *P* < 0.01), 0.734 (95% *CI* = 0.632–0.835, *P* < 0.001), and 0.706 (95% *CI* = 0.603–0.810, *P* < 0.01) for high risk of AIP, respectively.

**Conclusion:**

AIP has independent positive linear correlation with indicators related to overweight and obesity in FH patients; AIP has good predictive performance for overweight and obesity in FH patients, and WHR has good performance for identifying moderate and high risk of AIP in FH patients.

## Introduction

Familial hypercholesterolemia (FH) is an autosomal dominant disorder with an estimated prevalence of 1/300 to 1/500 in heterozygous populations and at least 20 million people worldwide with FH (1–5). It is well known that hypercholesterolemia predisposes to the formation of atherosclerotic plaques in the vascular wall and has a high risk of cardiovascular disease (CVD) events. The prevalence of FH is higher in patients who have experienced a CVD event, and control of other CVD risk factors appears to be less optimal than in other patients (6). The results of a cross-sectional survey of FH in China showed that the prevalence of FH in the Chinese population is similar to that in other countries; however, FH in China is mainly found in patients with early-onset coronary heart disease and their lipid levels are poorly controlled and at higher risk of CVD (7). Together with the fact that cholesterol is involved in the formation of cellular barriers for many basic physiological processes and acts as an important component of signal transduction (1), many studies have emphasized that patients with FH should be identified early and given early intervention (8–11).

The detection of traditional lipid profiles and their associated calculated indices are the main methods currently used to assess the risk of CVD. However, in the absence of an abnormal lipid profile, the possibility of coronary artery disease (CAD) cannot be excluded. Therefore, it has been suggested that different combinations of these lipid profile parameters could be used to identify such high-risk individuals. The atherogenic index of plasma (AIP), ApoB/ApoA1 Ratio (BAR) and the atherogenic coefficient (AC) have been considered as high-quality predictors of cardiovascular risk (12, 13). In recent years, AIP has gained widespread interest as a screening tool for dyslipidemia and is considered a major cardiometabolic risk factor and an emerging indicator to predict CVD risk (14), reflecting the balance between atherogenic and antiatherogenic factors in an integrated manner (15, 16). BAR has also been proposed to be better than LDL-C and superior to non-high-dense lipoprotein cholesterol (non-HDL-C) as a marker of CVD risk (17–20). This ratio has also been considered as a potential marker of cardiovascular risk because it can often be abnormal in the presence of normal conventional lipid levels (21). A study by Lu et al. indicated that BAR is a valid predictor of coronary heart disease risk in overweight and obese people (22). Another ratio index that is HDL-C dependent and has significance in predicting CAD risk is AC, calculated as [(TC-HDL-C)/HDL-C] (23). It has been demonstrated that AC reflects the atherogenic potential of the entire lipoprotein fraction profile and can be used for therapeutic management (12).

It is well known that overweight and obesity-related indicators, including body mass index (BMI), waist-to-height ratio (WHtR) and waist-to-hip ratio (WHR), are among the good criteria to reflect the degree of body fatness and healthiness, and are widely used to screen overweight and centrally obese people. A large epidemiological survey showed that more than two-thirds of deaths associated with high BMI were due to CVD (24). Abdominal obesity (also central obesity) involves the accumulation of abdominal fat and is considered an independent risk factor for obesity-related diseases and death (25). It has been reported that when AIP values of 0.12–0.21 and > 0.21 indicate the likelihood of critical abdominal obesity and abdominal obesity, respectively, while the combination of waist circumference and AIP may increase the specificity and sensitivity of abdominal obesity detection in clinical practice, thus suggesting that AIP may be used as a reference for estimating abdominal obesity (26).

In this study, to determine whether atherogenic lipid indicators such as AIP are independently associated with overweight and obesity-related indicators such as BMI, we analyzed the correlation between lipid parameters (i.e., lipid calculation indicators) such as AIP, BAR, and AC with overweight and obesity-related indicators, and finally evaluated the predictive performance of cardiovascular risk predictors for overweight and overweight and obesity-related indicators for AIP in risk and high risk identification performance. It is also hoped that these findings will highlight the threat of overweight and even obesity in FH patients and promote the benefits of weight control in FH patients, thus reducing the risk of atherogenic disease in this population.

## Patients and methods

### Inclusion of study subjects

The original data of the FH study samples were obtained from the subproject “Collection and clinical epidemiology of hereditary hyperlipidemia blood specimens in family lines” under the “Collection, preservation and utilization of genetic resources of major diseases” of the “Tenth Five-Year Plan” of China. The original data was initially screened from the population who attended the outpatient clinic of the First Hospital of Lanzhou University from 2004 to 2017 based on the initial fasting lipid levels, and then invited them and their first-degree relatives to undergo a physical examination again on a specified date, which included biochemical tests such as lipid panel, physical examination, electrocardiogram and face-to-face questionnaires, and all study subjects signed an informed consent form to voluntarily participate in this study. In addition, two of the following three criteria were met and included in the project: (1) At least two family members in each family line with dyslipidemia, as determined by the National Cholesterol Education Program (NCEP) (27), with TC ≥ 6.20 mmol/L and/or LDL-C ≥ 4.10 mmol/L without secondary causes; (2) at least 2 generations of involvement per family line; and (3) at least 1 member of each family line with hypercholesterolemia with an age of onset no older than 50 years.

**FIGURE 1:**
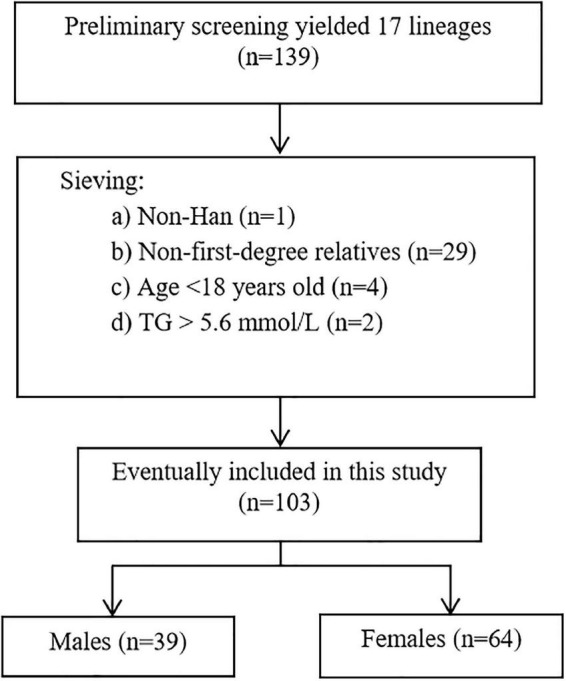
Flowchart of the study sample. FH, familial hypercholesterole mia; TG, triglycerides.

In order to make the above information meet the needs of the current study, we initially screened out cases in which the above information might bias the results, including non-Han, non-first-degree relatives, age < 18 years, and TG > 5.6 mmol/L. Finally, we followed the Dutch Lipid Clinic Network (DLCN) (28) for clinical lipid monitoring guidelines and included Patients with scores of 6 and above (i.e., definite FH and probable FH) were included in the current study the screening process for the study sample is shown in Figure 1.

### Questionnaire survey and clinical evaluation

#### Questionnaire

The questionnaire for the hyperlipidemia family blood specimen collection project was pre-designed by the researchers to obtain general demographic information, life and dietary habits, disease history and medication history of the participants. The survey involved in this study mainly included the following aspects: (1) general demographic information: such as ethnicity, gender, age, etc.; (2) lifestyle habits: such as smoking, alcohol consumption, physical exercise, etc.; (3) dietary habits: dietary preferences; (4) disease history: past history, current disease history and family history, etc.; (5) medication history: type of medication, name of medication, start date of medication, dose of medication, etc.

#### Clinical assessment

Clinical assessments were performed by uniformly trained clinicians. BMI was calculated by dividing weight (kg) by the square of height (m^2^), using an overweight cut-off point of 24 kg/m^2^ suitable for BMI in Asian populations (29), and the study population was divided into overweight and non-overweight groups. WHR was obtained by dividing waist circumference (cm) by hip circumference (cm), and WHtR calculated as waist circumference (cm)/height (cm.) WHtR ≥ 0.5 and WHR ≥ 0.9 in men and ≥ 0.85 in women were considered centrally obese (30). Xanthoma included tendinous xanthoma (which could be located the back of the fingers, elbows, knees, or elsewhere and also included the thickening of the Achilles tendon) and tuberous xanthoma, as well as rash and flat xanthoma. Fasting blood glucose (FBG) ≥ 7.0 mmol/L or those who had been treated with hypoglycemic therapy were identified as having diabetes. With regard to family history of disease, it was defined as a family history of appropriate disease in first-degree relatives (i.e., children, parents, and siblings) and grandparents in second-degree relatives of study subjects, including diabetes, hypertension, hypercholesterolemia, and CVD (coronary heart disease, stroke). Systolic blood pressure (SBP) ≥ 140 *mmHg* or diastolic blood pressure (DBP) ≥ 90 *mmHg* measured three times on non-same day or taking antihypertensive medication was defined as hypertension. Mean arterial pressure (MAP) was calculated as (SBP + 2DBP)/3. Those who smoked within the past 6 months and reached 1 cigarette/day were classified as smokers, those who never smoked or smoked occasionally but did not meet the smoking criteria or quit smoking for more than 1 year were classified as non-smokers. Those who drank alcohol continuously for more than 6 months and drank alcohol at least once a week on average were classified as alcohol drinkers, and those who never drank alcohol or drank occasionally but did not meet the criteria for drinking alcohol were classified as non-drinkers. The AIP risk was divided into three groups: (1) low risk, AIP < 0.11; (2) moderate risk, AIP ≥ 0.11 and ≤ 0.21; and (3) high risk, AIP > 0.21 (31, 32).

### Laboratory tests

The results of the laboratory analyses were obtained from the subjects’ data profiles. The analysis of biochemical items such as the full lipid panel was performed at the Laboratory Department of the First Hospital of Lanzhou University, and all blood sampling was performed on the following morning after 8–12 h of fasting, with appropriate quality control, using the same fully automated biochemical analyzer. Serum TC, HDL-C, LDL-C, and TG concentrations were measured by applying enzyme colorimetric method, serum ApoA1, ApoB and Lipoprotein (a) [Lp(a)] levels were measured by applying immunoturbidimetric method, and serum FBG levels were measured by applying hexokinase method. The above biochemical items were performed in an automated biochemical analyzer (Beckman Coulter, Brea, CA, USA). Based on independent lipid parameters, the following clinical indicators were calculated: non-HDL-C, AIP, BAR, LDL-C/ApoB ratio, HDL-C/ApoA1 ratio, LDL-C/HDL-C ratio, and AC. non-HDL-C values were obtained by subtracting HDL-C values from TC values. AIP was calculated as Log_10_ (TG/HDL-C) (15).

### Statistical analysis

Data were analyzed using SPSS version 20.0 (SPSS, Inc., Chicago, IL, USA). For continuous variables, normal distribution was expressed as mean ± standard deviation ($\bar{\text{x}}$ ± *s*) and non-normal distribution was expressed as median and quartiles [M (P25, P75)]; for categorical variables, it was expressed as number and percentage (N/%). Normality of continuous variables was tested using Shapiro-Wilk test and Q-Q plot test. In clinical characteristics and biochemical parameters between groups, for some physical and blood indicators such as MAP, BMI, WHR, which are normally distributed continuous variables, the Student’s *t*-test was applied to analyze the differences between two independent variables, and the chi-square test was performed before the *t*-test; for TG and Lp(a), which are two continuous variables that do not obey normal distribution, the Mann-Whitney *U*-test was applied to analyze the differences between two analysis of variance between independent variables. For the analysis of variance of categorical variables such as males, smokers, and alcohol drinkers, we applied the chi-square test. Pearson correlation analysis was used to determine the relationship between BMI, WHR, and WHtR and the levels of AIP, BAR, and AC. Multiple stepwise linear regression analysis was used to determine the independent correlations between BMI, WHR, and WHtR and the levels of AIP, BAR, and AC. Receiver operating characteristic (ROC) analysis was used to explore the performance of cardiovascular risk predictors in identifying overweight and central obesity. *P* < 0.05 was considered statistically significant, and all tests were two-sided.

## Results

### Basic information of familial hypercholesterolemia patients

After excluding samples with missing key data such as basic information, overweight and obesity-related indicators and lipid indicators, a total of 103 patients with FH from 17 family lines were finally included in this study. As shown in Table 1, the study subjects were all Han Chinese, including 39 (37.9%) males and 64 (62.1%) females, with an age range of 18–85 years, an overall mean age of (46.12 ± 14.29) years, and an overall mean BMI of (23.63 ± 3.39) kg/m^2^. The study subjects were classified according to BMI into overweight group (53, 51.4%) and non-overweight group (50, 48.6%), and the results were compared between the two groups for basic conditions showing age (*P* = 0.019), xanthoma (*P* = 0.027), hypertension (*P* < 0.001), MAP (*P* < 0.001), BMI (*P* < 0.001), WHR (*P* < 0.001), WHtR (*P* < 0.001), FBG (*P* = 0.012), TG (*P* = 0.002), HDL-C (*P* = 0.016), ApoA1 (*P* = 0.007), AIP (*P* = 0.001), BAR (*P* = 0.003), AC (*P* = 0.029), and LDL-C/HDL-C ratio (*P* = 0.035) were statistically significant between the two groups, while gender, smoking, alcohol consumption, dietary oiliness, dietary saltiness, history of coronary heart disease, TC, LDL-C, ApoB, Lp(a), non-HDL-C, LDL-C/ApoB, and HDL-C/ApoA1 were not statistically significant between the two groups (*P* > 0.05). The study subjects were divided into central obesity group (59 cases, 59%) and non-central obesity group (41 cases, 41%) according to WHR and WHtR, and the basic conditions were compared between the two groups, which showed that age (*P* = 0.001), male (*P* = 0.023), smoking (*P* = 0.024), xanthoma (*P* = 0.014), hypertension (*P* = 0.003), MAP (*P* < 0.001), BMI (*P* < 0.001), WHR (*P* < 0.001), WHtR (*P* < 0.001), FBG (*P* = 0.007), TG (*P* < 0.001), HDL-C (*P* = 0.010), ApoA1 (*P* = 0.007), and AIP (*P* < 0.001) were statistically significant between the two groups. statistically significant, whereas the differences in alcohol consumption, dietary oiliness, dietary salinity, history of coronary heart disease, TC, LDL-C, ApoB, Lp(a), non-HDL-C, LDL-C/ApoB, BAR, AC, LDL-C/HDL-C, LDL-C/ApoB, and HDL-C/ApoA1 were not statistically significant between the two groups (*P* > 0.05).

**TABLE 1:** Basic Clinical features of patients with familial hypercholesterolemia.

Variables	All *n* = 103	BMI ≥ 24 kg/m^2^		*P*	Central obesity		*P*
		No (*n* = 53)	Yes (*n* = 50)		No (*n* = 59)	Yes (*n* = 41)	
Age (years)	46.12 ± 14.29	42.94 ± 16.27	49.48 ± 11.04	**0.019**	42.19 ± 15.80	51.54 ± 10.24	**0.001**
Male (*N*/%)	39 (37.9%)	17 (32.1%)	22 (44%)	0.212	17 (28.8%)	21 (51.2%)	**0.023**
Smokers (*N*/%)	28 (27.2%)	12 (22.6%)	16 (32%)	0.286	11 (18.6%)	16 (39%)	**0.024**
Alcohol drinkers (*N*/%)	45 (43.7%)	23 (43.4%)	22 (44%)	0.286	26 (44.1%)	16 (39%)	0.615
Dietary oiliness (*N*/%)	21 (20.4%)	12 (22.6%)	9 (18%)	0.559	12 (20.3%)	9 (22%)	0.846
Salty diet (*N*/%)	27 (26.2%)	10 (18.9%)	17 (34%)	0.081	11 (18.6%)	15 (36.6%)	0.063
CHD history (*N*/%)	5 (4.8%)	2 (3.8%)	3 (6%)	0.599	3 (5.1%)	2 (4.9%)	0.925
Xathoma (*N*/%)	18 (17.5%)	5 (9.4%)	13 (26%)	**0.027**	6 (10.2%)	12 (29.3%)	**0.014**
Hypertension (*N*/%)	34 (33%)	8 (15.1%)	26 (52%)	**< 0.001**	12 (20.3%)	20 (48.8%)	**0.003**
MAP (mmHg)	91.24 ± 14.11	85.77 ± 12.40	97.03 ± 13.58	**< 0.001**	86.47 ± 12.49	97.19 ± 13.83	**< 0.001**
BMI (kg/m^2^)	23.63 ± 3.39	20.94 ± 1.91	26.48 ± 1.99	**< 0.001**	21.85 ± 2.72	25.97 ± 2.73	**< 0.001**
WHR	0.87 ± 0.08	0.83 ± 0.07	0.91 ± 0.08	**< 0.001**	0.82 ± 0.06	0.94 ± 0.06	**< 0.001**
WHtR	0.51 ± 0.06	0.47 ± 0.04	0.55 ± 0.04	**< 0.001**	0.47 ± 0.04	0.56 ± 0.04	**< 0.001**
FBG (mmol/L)	4.96 ± 0.79	4.77 ± 0.68	5.16 ± 0.85	**0.012**	4.78 ± 0.62	5.21 ± 0.93	**0.007**
TC (mmol/L)	5.86 ± 1.41	5.82 ± 1.21	5.92 ± 1.59	0.724	5.95 ± 1.27	5.74 ± 1.61	0.483
TG (mmol/L)	1.48 (0.90–2.34)	1.10 (0.76–1.88)	1.78 (1.10–2.61)	**0.002**	1.1 (0.77–1.72)	1.92 (1.38–2.78)	**< 0.001**
HDL-C (mmol/L)	1.33 ± 0.27	1.39 ± 0.28	1.26 ± 0.25	**0.016**	1.39 ± 0.26	1.25 ± 0.27	**0.010**
LDL-C (mmol/L)	3.95 ± 1.30	3.82 ± 1.12	4.08 ± 1.46	0.313	3.95 ± 1.20	3.90 ± 1.46	0.876
ApoA1 (g/L)	1.43 ± 0.27	1.50 ± 0.29	1.36 ± 0.24	**0.007**	1.49 ± 0.28	1.34 ± 0.25	**0.007**
ApoB (g/L)	0.93 ± 0.50	0.86 ± 0.22	0.91 ± 0.25	0.262	0.89 ± 0.25	0.87 ± 0.22	0.658
Lp(a) (mg/L)	249.5 (168–309)	227.5 (175–324)	256.5 (156–304)	0.905	238 (168–329)	256.5 (147–286)	0.762
AIP	0.05 ± 0.30	-0.05 ± 0.29	0.15 ± 0.28	**0.001**	−0.06 ± 0.27	0.21 ± 0.29	**< 0.001**
BAR	0.63 ± 0.17	0.58 ± 0.15	0.68 ± 0.18	**0.003**	0.61 ± 0.16	0.66 ± 0.19	0.103
AC	3.50 ± 1.17	3.26 ± 0.90	3.76 ± 1.35	**0.029**	3.35 ± 0.94	3.68 ± 1.34	0.168
LDL-C/HDL-C	3.05 ± 1.14	2.82 ± 0.92	3.28 ± 1.29	**0.035**	2.91 ± 0.96	3.19 ± 0.16	0.227
LDL-C/ApoB	4.49 ± 0.95	4.53 ± 1.04	4.44 ± 0.86	0.649	4.50 ± 1.03	4.42 ± 0.86	0.693
HDL-C/ApoA1	0.93 ± 0.12	0.93 ± 0.12	0.93 ± 0.13	0.911	0.94 ± 0.12	0.93 ± 0.13	0.845
Non-HDL-C (mmol/L)	4.54 ± 1.32	4.42 ± 1.11	4.65 ± 1.51	0.385	4.55 ± 1.19	4.49 ± 1.52	0.822

Data are presented as mean ± standard deviation, median (25th–75th percentile) or *n* (%). Bold values indicate statistical significance.

### Relationship between overweight and obesity-related indicators and cardiovascular risk predictors in patients with familial hypercholesterolemia

#### Simple correlation analysis

**FIGURE 2:**
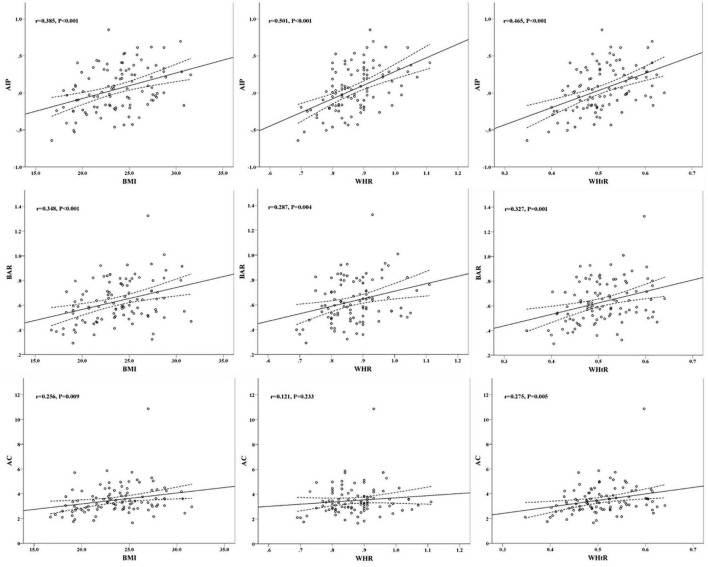
Scatter plots of correlation between overweight and obesity indicators and cardiovascular risk predictors.

Further analysis of linear relationships between overweight and obesity-related indicators and conventional lipid profiles and lipid-related calculated parameters in patients with FH, as shown in Table 2, revealed that BMI was significantly negatively correlated (*P* < 0.01) with HDL-C (*r* = −0.284) and ApoA1 (*r* = −0.269), and with AIP (*r* = 0.385), BAR (*r* = 0.348) and AC (*r* = 0.256) were significantly positively correlated (*P* < 0.01); WHR was significantly negatively correlated with HDL-C (*r* = −0.303) and ApoA1 (*r* = −0.361) (*P* < 0.01) and positively correlated with TG (*r* = 0.329), AIP (*r* = 0.501) and BAR (*r* = 0.287) (*P* < 0.01). WHtR showed significant negative correlations (*P* < 0.05) with HDL-C (*r* = −0.196) and ApoA1 (*r* = −0.203), and significant positive correlations (*P* < 0.01). The overall significant correlations of overweight and obesity-related indicators with AIP, BAR and AC among lipid parameters in FH patients were shown, and the scatter plots of correlations between BMI, WHR and WHtR and AIP, BAR, and AC were plotted in Figure 2.

**TABLE 2:** Correlation analysis of overweight and obesity indicators and lipid parameters in patients with familial hypercholesterolemia.

	BMI		WHR		WHtR	
	*r*	*P*	*r*	*P*	*r*	*P*
TC (mmol/L)	0.048	0.634	–0.112	0.272	0.136	0.173
TG (mmol/L)	0.233	0.018	0.329	**0.001**	0.310	**0.002**
HDL-C (mmol/L)	–0.284	**0.004**	–0.303	**0.002**	–0.196	**0.048**
LDL-C (mmol/L)	0.126	0.207	–0.017	0.865	0.216	**0.029**
ApoA1 (g/L)	–0.269	**0.006**	–0.361	**< 0.001**	–0.203	**0.041**
ApoB (g/L)	0.161	0.105	0.031	0.760	0.186	0.061
Lp(a) (mg/L)	–0.058	0.689	0.035	0.810	–0.069	0.633
AIP	0.385	**< 0.001**	0.501	**< 0.001**	0.465	**< 0.001**
BAR	0.348	**< 0.001**	0.287	**0.004**	0.327	**0.001**
AC	0.256	**0.009**	0.121	0.233	0.275	**0.005**
LDL-C/HDL-C	0.247	0.012	0.123	0.224	0.282	0.004
LDL-C/ApoB	–0.059	0.554	–0.128	0.206	0.022	0.823
HDL-C/ApoA1	–0.050	0.615	0.056	0.584	–0.009	0.929
Non-HDL-C (mmol/L)	0.109	0.274	–0.057	0.577	0.185	0.062

Pearson correlation analyses were used. Bold values indicate statistical significance.

#### Independent correlation analysis

Given the above correlations, independent correlation analyses between BMI, WHR and WHtR and AIP, BAR and AC were performed by applying multiple stepwise linear regression, as shown in Tables 3–5. After adjusting for age, sex, smoking, xanthoma, MAP and FBG, the results showed that independent correlations with BMI were AIP (β = 0.020, *P* = 0.013) and BAR (β = 0.015, *P* = 0.003), AIP (β = 1.176, *P* = 0.001) independently associated with WHR, and AIP (β = 1.575, *P* = 0.001), BAR (β = 0.661, *P* = 0.024) and AC (β = 0.427, *P* = 0.035) independently associated with WHtR. It can be seen that overweight and obesity-related indicators BMI, WHR, and WHtR all had independent positive linear correlations with AIP.

**TABLE 3:** Independent correlation analysis of cardiovascular risk predictors with BMI.

	BMI (un-adjusted)			BMI (adjusted)		
	Constant	β	*P*	Constant	β	*P*
AIP	–0.765	0.035	**< 0.001**	−1.169	0.020	**0**.**013**
BAR	0.210	0.018	**< 0.001**	0.138	0.015	**0**.**003**
AC	1.412	0.088	**0.009**	/^a^	/	/

Multivariable stepwise linear regression models are shown. Adjusted confounders included age, sex, smoking, xanthoma, MAP and FBG. Bold values indicate statistical significance. ^a^“/”denotes no independent correlation.

**TABLE 4:** Independent correlation analysis of cardiovascular risk predictors with WHR.

	WHR (un-adjusted)			WHR (adjusted)			
	Constant	β	*P*	Constant	β	*P*
AIP	–1.557	1.848	**< 0.001**	−1.692	1.176	**0.001**
BAR	0.105	0.605	**0.004**	/	/	/
AC	1.991	1.716	0.233	/	/	/

Multivariable stepwise linear regression models are shown. Adjusted confounders included age, sex, smoking, xanthoma, MAP and FBG. Bold values indicate statistical significance.

**TABLE 5:** Independent correlation analysis of cardiovascular risk predictors with WHtR.

	WHtR (un-adjusted)			WHtR (adjusted)			
	Constant	β	*P*	Constant	β	*P*
AIP	–1.124	2.314	**< 0.001**	–0.967	1.575	**0.001**
BAR	0.160	0.927	**0.001**	0.046	0.661	**0.024**
AC	0.841	5.244	**0.005**	1.288	0.427	**0.035**

Multivariable stepwise linear regression models are shown. Adjusted confounders included age, sex, smoking, xanthoma, MAP and FBG. Bold values indicate statistical significance.

### Predictive performance analysis of cardiovascular risk predictors for overweight and obesity in patients with familial hypercholesterolemia

**FIGURE 3:**
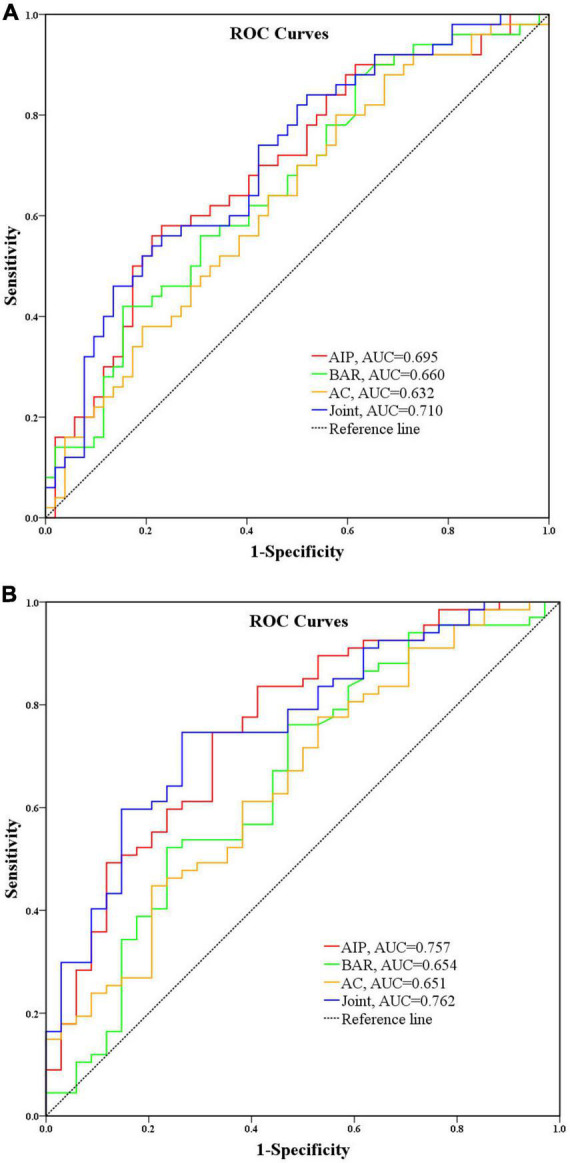
**(A)** Predictive performance analysis of AIP, BAR, and AC for overweight. **(B)** Predictive performance analysis of AIP, BAR, and AC for central obesity.

To further assess the role of AIP, BAR and AC in identifying overweight as well as central obesity conditions in FH patients, we plotted ROC curves, which showed that the area under the ROC (AUC) for overweight when AIP, BAR, AC and combined triple indicators were 0.695 (95% *CI* = 0.593–0.797, *P* = 0.001), 0.660 (95% *CI* = 0.555–0.766, *P* = 0.005), 0.632 (95% *CI* = 0.525–0.740, *P* = 0.021) and 0.710 (95% *CI* = 0.611–0.810, *P* < 0.001), respectively, as shown in Figure 3A; the AUCs for central obesity with AIP, BAR and AC and the combination of all three were 0.757 (95% *CI* = 0.656–0.857, *P* < 0.001), 0.654 (95% *CI* = 0.536–0.771, *P* = 0.012), 0.651 (95% *CI* = 0.538–0.764, *P* = 0.013) and 0.762 (95% *CI* = 0.666–0.858, *P* < 0.001), Figure 3B. It can be seen that AIP has the best predictive performance for overweight and obesity among cardiovascular risk predictors, while the area under the curve suggests the possibility that AIP its predictive performance for obesity is better than that for overweight; in addition, the combined AIP, BAR, and AC three indicators have a moderate predictive performance for overweight in FH patients.

### Analysis of the identification performance of overweight and obesity-related indicators in familial hypercholesterolemia patients for moderate and high risk of atherogenic index of plasma

**FIGURE 4:**
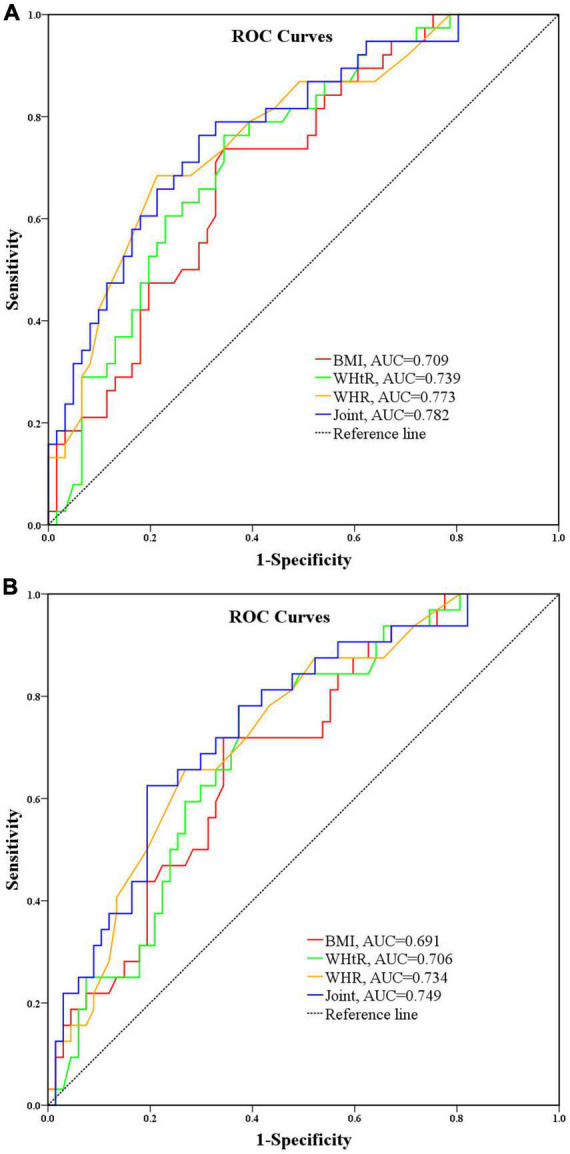
**(A)** Performance analysis of BMI, WHR, and WHtR for identifying moderate risk of AIP. **(B)** Performance analysis of BMI, WHR, and WHtR for identifying high risk of AIP.

AIP is known to have the best predictive performance for overweight and obesity based on BMI, WHR and WHtR judgments, however, to explore which indicator is more accurate for identifying the risk level of AIP, the AUC was further used to compare the three overweight and obesity related indicators for identifying moderate risk of AIP and high risk of AIP, respectively, and the results showed that the AUC for BMI, WHR, and WHtR for moderate risk were 0.709 (95% *CI* = 0.608–0.811, *P* < 0.001), 0.773 (95% *CI* = 0.678–0.867, *P* < 0.001), and 0.739 (95% *CI* = 0.641–0.836, *P* < 0.001), respectively, as shown in Figure 4A; the AUCs of BMI, WHR, and WHtR for AUC for high risk of AIP were 0.691 (95% *CI* = 0.585–0.797, *P* = 0.002), 0.734 (95% *CI* = 0.632–0.835, *P* < 0.001), and 0.706 (95% *CI* = 0.603–0.810, *P* = 0.001), respectively, as shown in Figure 4B. It can be seen that the three overweight and obesity-related indicators BMI, WHR, and WHtR have good identification performance for both moderate and high risk of AIP, with WHR having the largest AUC, followed by WHtR, and BMI having the smallest. It is suggested that WHR has better and more robust performance in identifying moderate and high risk of AIP. As for the combined diagnostic effectiveness, the AUC of combining BMI, WHR, and WHtR was 0.782 (95% *CI* = 0.689–0.874, *P* < 0.001) for moderate risk of AIP and 0.749 (95% *CI* = 0.648–0.850, *P* < 0.001) for high risk of AIP. It showed that the combination of BMI, WHR, and WHtR had a moderate level of discrimination ability for moderate and high risk of AIP although the discrimination performance was not significantly improved compared to the individual indicators.

## Discussion

In this study, by analyzing the correlation between cardiovascular risk predictors and overweight and obesity-related indicators in patients with FH, the results showed that AIP was independently associated with BMI, WHR and WHtR, BAR was independently associated with BMI and WHtR, and AC was independently associated with WHtR. Although independent correlations between AIP and BMI have been reported (33) and between BAR and waist circumference (34), up to now, in patients with FH, the present study is the first to report correlations regarding the group of cardiovascular risk predictors in patients with FH with the group of overweight and obesity-related indicators. The significance of this study is that (1) by analyzing the correlation between cardiovascular risk predictors and overweight and obesity-related indicators in patients with FH families, it provides a theoretical basis for actively controlling overweight and obesity-related indicators in FH patients and thus reducing CVD risk, which has important public health implications; (2) among cardiovascular risk predictors, AIP was found to have the strongest predictive effect on overweight, especially central obesity, which provides a basis for identifying overweight, especially obesity, through cardiovascular risk predictors.

AIP is a more comprehensive indicator of the balanced relationship between atheroprotective and atherogenic factors than a simple lipid index. The results of this study also showed that AIP among cardiovascular risk predictors has a better performance than BAR and AC both in terms of independent correlation with overweight and obesity-related indicators and in terms of identification of overweight and obesity. In fact, Shen et al. have reported that AIP can be a valid indicator for the assessment of abdominal obesity (26), and a recent cross-sectional study from a Chinese population also concluded that AIP was a novel and good biomarker associated with abdominal obesity (35). This is consistent with the results of the ROC curve analysis in the present study. Given the superiority of AIP over lipid indices alone, coupled with the fact that it can be easily calculated from conventional lipid profiles, AIP has gradually been increasingly favored and used in clinical practice to guide the assessment of CVD risk and disease prognosis in recent years (16, 13, 36–38). Therefore, it is reasonable to assume that CVD risk is further elevated in those obese populations in FH patients by the analysis of this study. As for the study on AIP in FH, Tomáš Freiberger’s team compared the levels of lipid-related parameters between FH patients with and without a history of CVD and found that AIP and TG were significantly higher in those with CVD events in FH, concluding that AIP is associated with a history of CVD in FH patients, which reflects the atherosclerotic small LDL and small HDL particles presence, which may be associated with the risk of CVD in FH patients (34). The results of the present study showed significantly higher TG and AIP as well as significantly lower HDL-C in overweight individuals with FH, suggesting an imbalance between atherogenic and anti-atherogenic factors. Moreover, in further multifactorial regression analysis in this study, BMI was independently associated with AIP, and BMI was an independent risk factor for increased risk level of AIP, suggesting that overweight patients with FH are at higher risk of CVD (33). It is well known that the main clinical manifestations of FH patients are significantly elevated atherogenic lipid indicators LDL-C and TC, and CVD events are the main cause of death in FH patients, and if BMI, an indicator associated with overweight and obesity, is not effectively controlled in these patients, it will add to their high CVD risk.

BAR represents the balance between ApoB-rich atherogenic particles and ApoA1-rich anti-atherogenic particles (17, 39) and is also considered as a potential marker of cardiovascular risk due to the fact that this ratio can often be abnormal in the presence of normal conventional lipid levels (21). It is generally accepted that a BAR above 0.9 is associated with a high risk of CVD (40), along with higher TG levels, AIP values and lower HDL-C levels (17). Showing that BAR was significantly and positively correlated with AIP, which is also consistent with what we observed in our results, some studies have also pointed out that AIP reflects the qualitative composition of lipoproteins, while BAR shows their quantity. Since they have different but complementary emphases, we suspect that they are intrinsically linked and hypothesize that there should be consistency in the manifestation of some diseases, and that combining these two indices to predict certain diseases may be promising. Currently, although the relationship between BMI and BAR is unclear, the results of some studies suggest that there may be an intrinsic association between BMI and BAR. A cohort study from China showed that both BMI and BAR were significantly elevated in patients with lactinoma (41), suggesting that there may be some intrinsic association between BMI and BAR in the disease state. Consistent with this, the results of the present study showed that BMI was independently associated with BAR in FH patients, suggesting that BMI is an independent influence on the elevated BAR in FH patients, and the present study also found that BAR indicators were significantly higher in overweight individuals than in non-overweight individuals, suggesting that overweight factors further increase the risk of CVD events in FH patients.

Several other lipid-related parameters, including non-HDL-C (42), AC (12), LDL-C/HDL-C (43), LDL-C/ApoB ratio (44, 45), and HDL-C/ApoA1 ratio (46, 47), have also been reported to be associated with CVD risk. However, the results of the current study did not show an independent correlation between BMI and these indicators. Although the results of the present study showed a significant correlation between BMI and LDL-C/HDL-C and AC in patients with FH, the correlation between them was found to disappear after adjusting for confounding factors.

It is generally accepted that age, male and blood pressure are important risk factors for cardiovascular events, and this is also true in patients with FH. Consistently, the results of the current study also showed that AIP was significantly correlated with age, male and MAP in addition to BMI independently, suggesting a higher risk of CVD in men with FH than in women, and the possibility that blood pressure is also a risk factor for CVD in patients with FH (27, 36, 48).

The results of this study showed that both AIP and BAR had significant independent correlations for BMI. However, by plotting ROC curves, it was shown that AIP was slightly better than BAR in predicting overweight. Our results also showed that cardiovascular risk predictors AIP, BAR, and AC were all independently correlated with WHtR among overweight and obesity-related indicators, but comparative analysis of ROC curves revealed that AIP was the strongest identifier of central obesity among the three cardiovascular risk predictors. Consistent with this result, one study found that AIP was significantly associated with BMI but not BAR by analyzing changes in cardiometabolic markers in overweight/obese children before and after lifestyle interventions; their results also showed that AIP was strongly associated with obesity, whereas BAR was not significantly associated with obesity (49). Although AIP is a calculated value, it is a sensitive indicator of dyslipidemia and may indirectly reflect the diameter of LDL-C particles (50). Therefore, we hypothesized that the combination of BMI and AIP could increase the specificity and sensitivity of overweight and even obesity detection in clinical practice. From Shen et al. showed that an AIP of 0.11–0.21 or > 0.21 suggested the possibility of borderline abdominal obesity or abdominal obesity, respectively, by examining the relationship between waist circumference and AIP, suggesting that AIP can be used as a reference for estimating abdominal obesity (26). Similarly, our results show a linear correlation between BMI and AIP, according to our derived mathematical expression for the relationship between AIP and BMI, an increase in BMI of 1.0 kg/m^2^ causes an increase in AIP of 0.035, an AIP value of 0.110 when BMI is 25 kg/m^2^, and an AIP ≥ 0.215 when BMI ≥ 28 kg/m^2^, which is essentially consistent with BMI ≥ 25 and ≥ 28 kg/m^2^ correspond to moderate risk (≥ 0.11) and high risk (≥ 0.21) for AIP, respectively, which also indicates that moderate risk AIP indicates overweight, while high risk AIP indicates the presence of obesity. WHtR ≥ 0.5 and/or WHR ≥ 0.9 in men and ≥ 0.85 in women are known to be considered centrally obese (30). According to the mathematical expression of the present study results AIP and WHR, AIP = 1.848WHR-1.557, bringing the AIP values of 0.11 and 0.24 into the formula, the resulting WHR values are 0.90 and 0.97, respectively, indicating that central obesity judged based on WHR corresponds to a moderate risk of AIP, and when WHR exceeds 0.97, patients with FH are at high risk of AIP. According to the mathematical expression of AIP and WHtR of the results of this study, AIP = 2.314WHtR-1.124, bringing the AIP values of 0.11 and 0.24 into the formula, the resulting WHtR values are 0.53 and 0.59, respectively, and it can be basically concluded that central obesity judged based on WHtR corresponds to moderate risk of AIP, and when WHtR exceeds 0.59 FH patients would have a high risk of AIP. Thus, it can be seen that if obesity judged based on BMI has a high risk of AIP, while central obesity judged based on WHtR and WHR has a moderate risk of AIP. On the other hand, the assessment of AIP risk based on BMI may be more sensitive than the assessment of AIP risk based on WHtR and WHR. However, the AIP risk level corresponding to obesity judged based on BMI and the AIP risk level corresponding to central obesity judged based on WHR combined with WHtR derived from this study contradict each other. In view of this, which of BMI, WHtR and WHR identifies the more reliable AIP risk, the present study again compared the identification of these three overweight and obesity-related indicators for intermediate AIP risk and high AIP risk, respectively, using AUC, and the results showed that WHR had the largest AUC, WHtR the second largest, and BMI the smallest for both intermediate AIP risk and high AIP risk. It is suggested that WHR may be a better and more robust identifier of overweight and obesity-related indicators for moderate and high risk of AIP.

However, there are still some shortcomings in this study: (1) Although our conclusions were obtained based on retrospective data analysis, the causal relationship between BMI and cardiovascular risk predictors such as AIP has not been clearly answered in this study, and deeper mechanisms based on genetic diagnosis need to be further explored. (2) With the accelerated urbanization in China, the increase of “small family” has made the collection of FH family cases more difficult. Although the sample size of this study is eligible for this small incidence genetic disease, a larger sample size study is still necessary to improve the robustness of the results and the reliability of the conclusions.

## Conclusion

(1) Overweight and obesity-related indicators BMI, WHR and WHtR in FH patients all had independent positive linear correlations with AIP; (2) among cardiovascular risk predictors, AIP has better performance for predicting overweight and obesity; (3) overweight and obesity-related indicators had better performance in identifying both medium and high risk for AIP, among which WHR had the best performance in identifying medium and high risk for AIP in patients with FH.

## Data availability statement

The data analyzed in this study is subject to the following licenses/restrictions: The data presented in this study are available on reasonable request from the corresponding author. Requests to access these datasets should be directed to JH, hejch@lzu.edu.cn.

## Ethics statement

Written informed consent was obtained from the individual(s) for the publication of any potentially identifiable images or data included in this article.

## Author contributions

JH: conceptualization, data curation, funding acquisition, project administration, resources, and supervision. YW: formal analysis, investigation, methodology, software, and writing—original draft. JH and YW: validation and writing—review and editing. Both authors contributed to the article and approved the submitted version.
